# Net primary productivity and its partitioning in response to precipitation gradient in an alpine meadow

**DOI:** 10.1038/s41598-017-15580-6

**Published:** 2017-11-09

**Authors:** Fangyue Zhang, Quan Quan, Bing Song, Jian Sun, Youjun Chen, Qingping Zhou, Shuli Niu

**Affiliations:** 1 Key Laboratory of Ecosystem Network Observation and Modeling, Institute of Geographic Sciences and Natural Resources Research, CAS, Beijing, 100101 China; 2 University of Chinese Academy of Sciences, No.19 A Yuquan Road, Beijing, 100049 China; 3 State Key Laboratory of Vegetation and Environmental Change, Institute of Botany, CAS, Beijing, 100093 China; 4 Institute of Qinghai-Tibetan Plateau, Southwest University for Nationalities, Chengdu, 610041 China

## Abstract

The dynamics of net primary productivity (NPP) and its partitioning to the aboveground versus belowground are of fundamental importance to understand carbon cycling and its feedback to climate change. However, the responses of NPP and its partitioning to precipitation gradient are poorly understood. We conducted a manipulative field experiment with six precipitation treatments (1/12 P, 1/4 P, 1/2 P, 3/4 P, P, and 5/4 P, P is annual precipitation) in an alpine meadow to examine aboveground and belowground NPP (ANPP and BNPP) in response to precipitation gradient in 2015 and 2016. We found that changes in precipitation had no significant impact on ANPP or belowground biomass in 2015. Compared with control, only the extremely drought treatment (1/12 P) significantly reduced ANPP by 37.68% and increased BNPP at the depth of 20–40 cm by 80.59% in 2016. Across the gradient, ANPP showed a nonlinear response to precipitation amount in 2016. Neither BNPP nor NPP had significant relationship with precipitation changes. The variance in ANPP were mostly due to forbs production, which was ultimately caused by altering soil water content and soil inorganic nitrogen concentration. The nonlinear precipitation-ANPP relationship indicates that future precipitation changes especially extreme drought will dramatically decrease ANPP and push this ecosystem beyond threshold.

## Introduction

The terrestrial ecosystem has experienced frequent and extreme precipitation events during the last five decades^1–5^, which is projected to become even more frequent and severe during the remainder of the 21^st^ century^6,7^. Because precipitation is a primary determinant of plant growth, its variation has profound impacts on net primary productivity (NPP) of the terrestrial ecosystems^8,9^. Thus, a robust understanding of the relationship between precipitation and NPP is critical but a big challenge for better prediction of carbon cycle in response and feedback to climate change^10^.

The precipitation-NPP relationship has been studied by spatial approach, temporal approach, and manipulative experiments. Spatial approach basically uses precipitation transect to relate aboveground NPP (ANPP) with precipitation changes along a precipitation gradient. The spatial models mostly show that ANPP increases linearly with mean annual precipitation in meadow steppes^11^, temperate grasslands^12^ and alpine grasslands^13^. The temporal studies relate time series of ANPP and annual precipitation in a single site and also find linear relationship between them but with lower slopes and regression coefficients than spatial models^14,15^. Because the constraint of plant communities and soil biogeochemistry, temporal models in a single site are more preferred over spatial models to forecasts precipitation effects on ANPP^14^. Recently, Knapp, *et al*.^16^ proposed a double asymmetry hypothesis, which used a nonlinear model to fit precipitation-ANPP relationship. Specifically, when spanning large gradients in precipitation or in extreme precipitation years, the relationship of ANPP and precipitation will display a positive or negative asymmetry. However, few studies are conducted to test or support this nonlinear relationship^17,18^. Although some manipulative experiments have been set up to examine the relationship between precipitation and ANPP, the relationship is restricted by the limited range of rainfall that mostly have two or three levels of precipitation treatments^19^. To gain empirical evidence of ANPP responses to large variations in precipitation, it is imperative to conduct field precipitation gradient experiments, with multiple levels of precipitation, especially the extreme precipitation condition.

Compared with ANPP, belowground production is even less understood, largely owing to the methodological difficulties of observation and measurement of root biomass^20^. In grasslands, belowground production contributes more than half of total primary production and is the major input of organic matter into soil^21,22^. Therefore, understanding the relationship of belowground production and precipitation is crucial to improve our knowledge of NPP variability in response to future global precipitation regimes. There are a few studies on the responses of belowground biomass (BGB) to precipitation change, but generate large debates. For example, a transect study in the Inner Mongolia grassland showed a linear relationship of BGB with precipitation gradient of 170 mm to 370 mm^23^. Nevertheless, a transect study along a precipitation gradient from 430 mm to 1200 mm in the Great Plains found that BGB were largely constant^12^. Only a few manipulative experiments were conducted to examine belowground NPP (BNPP) response to precipitation changes^24–26^, but none of them studied the response to a precipitation gradient.

The partitioning of BNPP associated with ANPP, commonly defined as *f*_BNPP_, is a critical variable reflecting plant growth strategy under changing environmental conditions^27,28^. *f*_BNPP_ is also a crucial parameter of terrestrial ecosystem carbon modeling, providing important constraints on the calibration and testing of dynamic carbon-cycling models^29,30^. Based on ‘functional equilibrium’ of biomass allocation, plants are assumed to allocate more biomass towards roots under limited water condition^31^. However, due to the limited studies on BNPP, how *f*_BNPP_ would respond to precipitation gradient is highly uncertain.

Responses of ANPP and BNPP to precipitation changes can be attributable to changes in abiotic factors of soil water content, soil temperature, and soil available nitrogen^32–34^ and the biotic changes in species composition or carbon allocations. Soil has complicated physical and biological characteristics, which will determine the water holding capacity and thus influence water availability that not necessarily reflects precipitation changes^35^. Meanwhile, precipitation changes will influence soil temperature through changing soil evaporation and plant transpiration^36^. Water addition usually decreases soil temperature due to soil moisture increase^37^. In addition, rate of nitrogen mineralization is higher in wet than dry condition, leading to changes in soil nitrogen availability^38,39^. Moreover, different plant functional types have various sensitivities to precipitation changes^32^, thus species composition influences NPP response as well. However, how these processes or mechanisms play roles along precipitation gradient are not well quantified or understood yet in specific studies.

The Tibetan Plateau is one of the most sensitive areas in response to global climate change^40,41^. Precipitation strongly determines NPP variations in this area because precipitation gradient characterizes not only vegetation distribution but also soil nitrogen conditions^42^. In a transect study in the Tibetan grasslands, both aboveground biomass and belowground biomass were positively correlated with soil moisture^43^. A temporal study in southeast of Tibetan Plateau also showed ANPP was linearly correlated with annual precipitation across years^44^. However, few studies have been done to examine responses of NPP and its partitioning along a precipitation gradient in Tibetan Plateau. In this study, by using a precipitation gradient experiment, we studied responses of ANPP, BNPP and *f*_BNPP_ to precipitation changes. Specifically, we addressed the following questions: (1) How does ANPP, BNPP and *f*_BNPP_ respond to changes in precipitation gradient in an alpine meadow? (2) What are the key factors controlling the responses of NPP and its partitioning to precipitation changes?

## Results

### Precipitation and Soil water content

**Figure 1:**
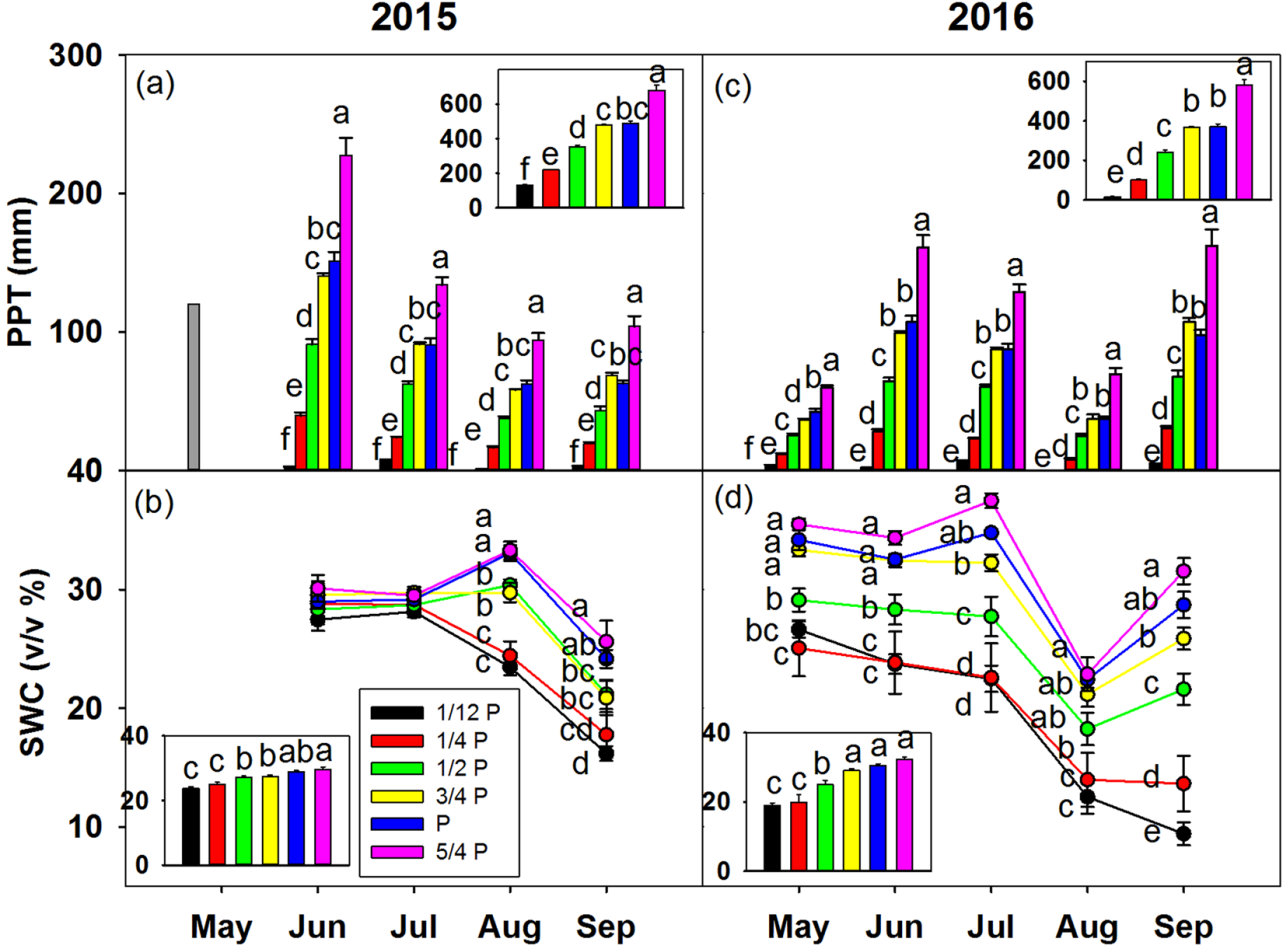
Treatment-induced changes in monthly precipitation (PPT, mm/yr) (**a**) and soil water content (SWC, v/v %) at the depth of 10 cm (**b**) from June to September 2015, and monthly PPT (**c**) and SWC (**d**) from May to September 2016. Inserted figure in panel shows the average values of variables under six levels over the growing season, values are mean ± SE. Different letters indicate statistically significant difference between treatments at *P* < 0.05.

Ambient precipitation over the entire growing season (from May to September) in our study site changed from 132.74 ± 0.69 mm in 1/12 P treatment to 679.54 ± 28.49 mm in 5/4 P treatment in 2015, and from 15.45 ± 1.36 mm in 1/12 P treatment to 581.22 ± 26.61 mm in 5/4 P treatment in 2016 (Fig. 1a,c).

Rainfall manipulation caused significant changes in soil water content (SWC) until August 2015. The average SWC over the growing season in 2015 ranged from 23.81 ± 0.49% in 1/12 P treatment to 29.62 ± 0.79% in 5/4 P treatment (*P* < 0.0001, Fig. 1b). In 2016, treatments had significant effect on SWC, throughout the whole growing season (*P* < 0.0001, Fig. 1d). The average SWC in 2016 ranged from 18.95 ± 0.78% under 1/12 P treatment to 32.32 ± 0.66% under 5/4 P treatment. Soil temperature was not significantly changed by the treatments, but the soil inorganic nitrogen (SIN) changed from 12.98 ± 1.31 mg L^−1^ under 1/12 P treatment to 19.56 ± 3.00 mg L^−1^ under 5/4 P treatment.

### Precipitation effects on ANPP, BGB, BNPP and *f*_BNPP_

**Figure 2:**
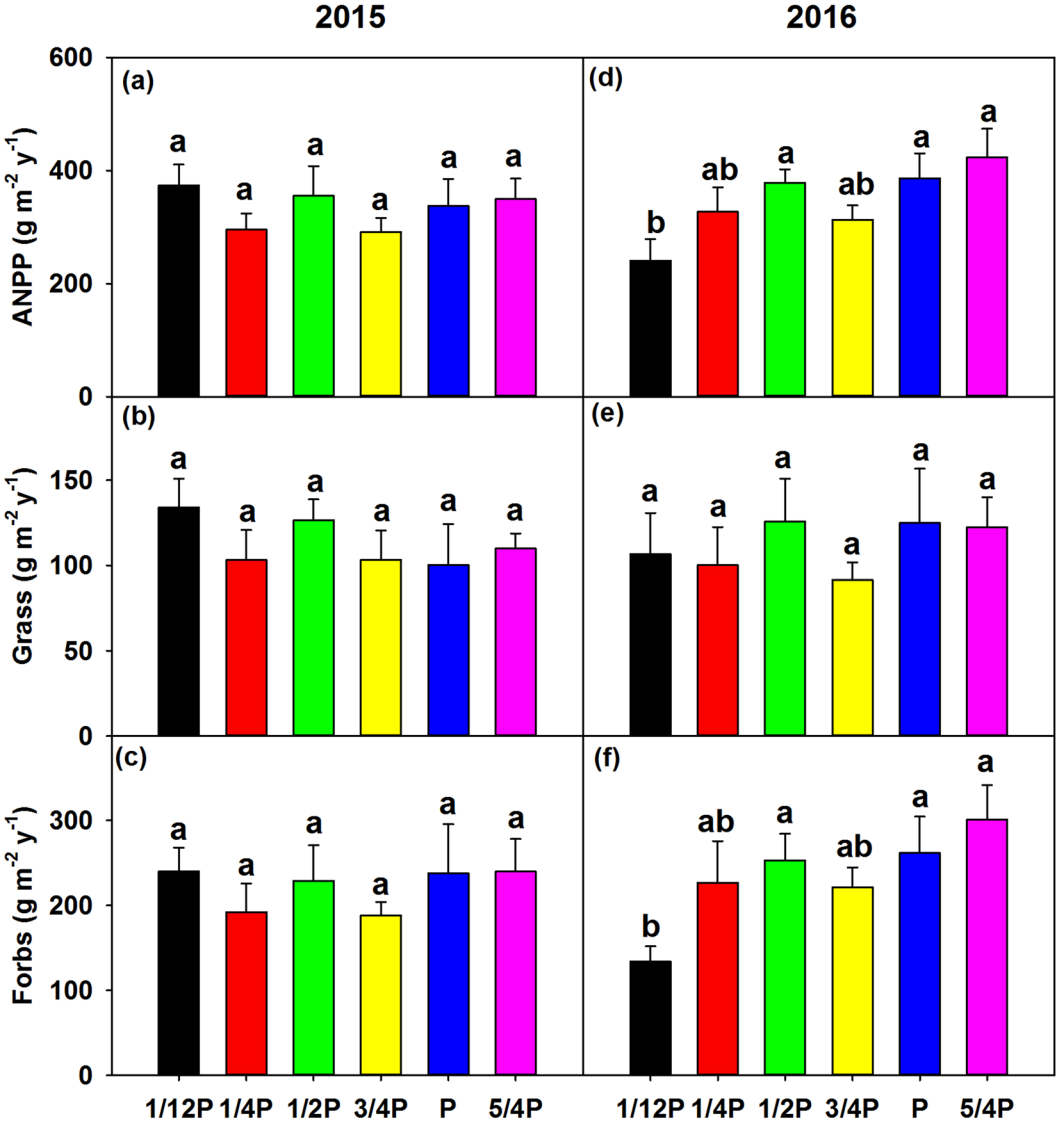
Variation in aboveground net primary productivity (ANPP) (**a**,**d**), and ANPP of grass (**b**,**e**) and forbs (**c**,**d**) under treatments in 2015 and 2016, values are mean ± SE. Different letters indicate statistically significant difference between treatments at *P* < 0.05.

In 2015, ANPP didn’t vary significantly among treatments (Fig. 2a). However, it significantly varied from 240.80 ± 37.94 g m^−2^ y^−1^ under 1/12 P treatment to 423.08 ± 50.77 g m^−2^ y^−1^ under 5/4 P treatment in 2016 (*P* < 0.05, Fig. 2d). ANPP was reduced by 37.68% (*P* = 0.01) under 1/12 P treatment in 2016. When separating aboveground biomass into different plant functional types, differential responses between grasses and forbs were observed along the precipitation gradient. The precipitation treatments marginally impacted biomass of forbs (*P* = 0.06), but not on grasses (*P* = 0.84) in 2016 (Fig. 2f). The lowest forbs biomass was 134.13 ± 17.59 g m^−2^ y^−1^ under 1/12 P treatment, and the highest one was 300.61 ± 40.88 g m^−2^ y^−1^ under 5/4 P treatment. Neither grasses nor forbs biomass was significantly impacted by precipitation gradient in 2015 (Fig. 2b,c).

**Figure 3:**
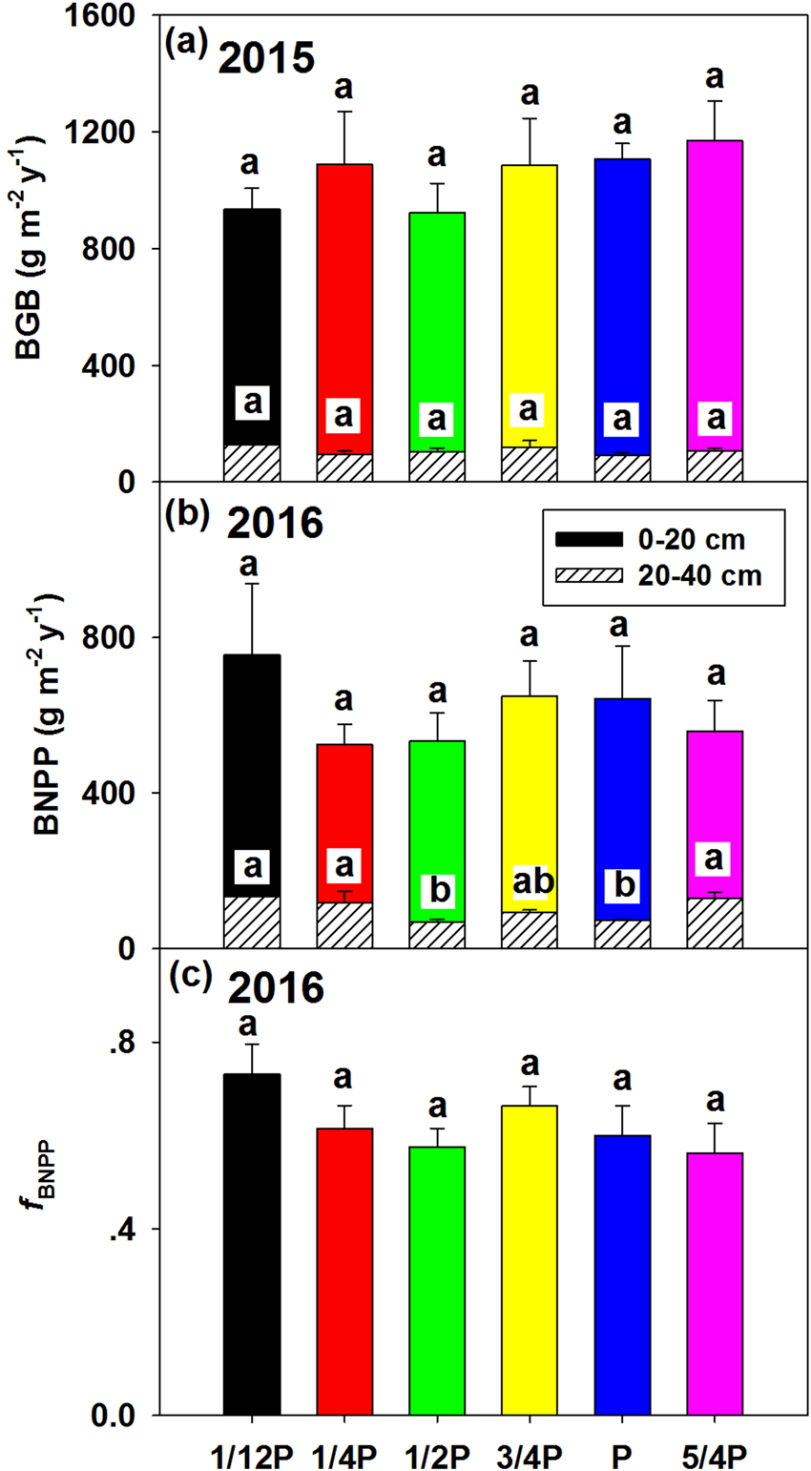
Variation in belowground biomass (BGB) under treatments in 2015 (**a**), and variation in belowground net primary productivity (BNPP) and *f*_BNPP_ in 2016 (**b**,**c**). Open bars in a, b indicate BGB or BNPP at the depth of 0–20 cm, hatched bars indicate BGB or BNPP at the depth of 20–40 cm, values are mean ± SE.

No significant effect of precipitation on BGB was observed in 2015 (*P* = 0.69, Fig. 3a). In 2016, the 1/12 P plots tended to have the highest BNPP and *f*_BNPP_ among precipitation treatments (Fig. 3b,c). The treatments significantly changed BNPP at the depth of 20–40 cm in 2016 (*P* = 0.01; Fig. 3b). Specifically, BNPP at 20–40 cm was increased by 80.59% under 1/12 P treatment, 58.75% under 1/4 P treatment and 74.43% under 5/4 P treatment, respectively. However, roots at 20–40 cm only accounted for 7.25% and 11.54% of the total BGB and BNPP, respectively. Thus, total BGB or BNPP at 0–40 cm was not significantly changed by precipitation treatments.

### Relationships of productivity with precipitation amount

**Figure 4:**
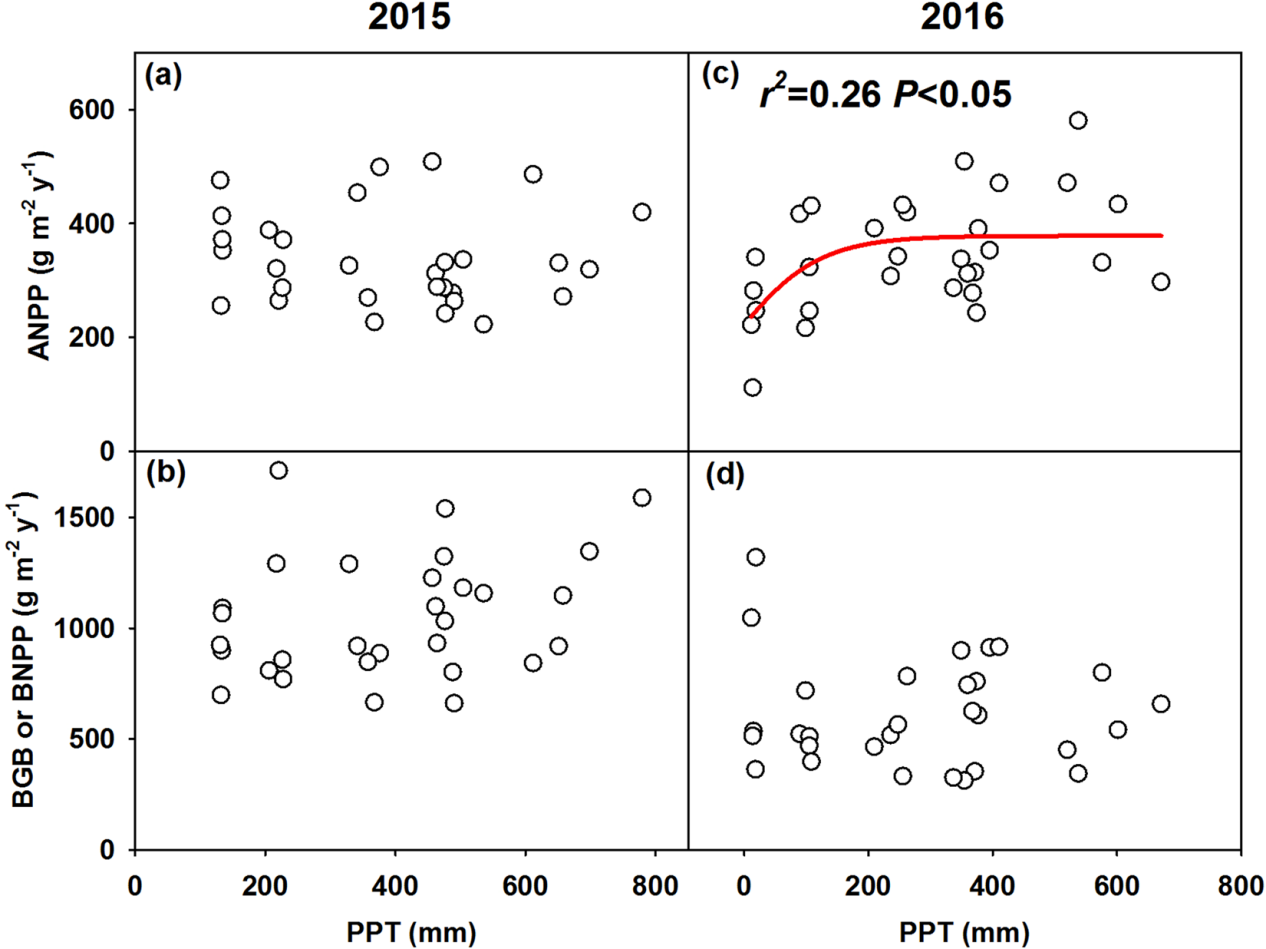
Relationships of above-ground net primary productivity (ANPP), belowground biomass (BGB) and below-ground net primary productivity (BNPP) with growing season precipitation of treatments (PPT) in 2015 (**a**,**b**) and 2016 (**c**,**d**). Nonlinear model, ANPP = 378.33/(1 + exp(−(PPT + 23.73)/68.82).

There was no significant relationship between precipitation and ANPP across plots in 2015 (Fig. 4a). However, ANPP increased nonlinearly with increasing precipitation in 2016 (*P* = 0.02, *r* ^2^ = 0.26; Fig. 4c). There was no significant relationship of BGB or BNPP with precipitation in either year (Fig. 4b,d).

### Factors controlling ANPP changes

**Figure 5:**
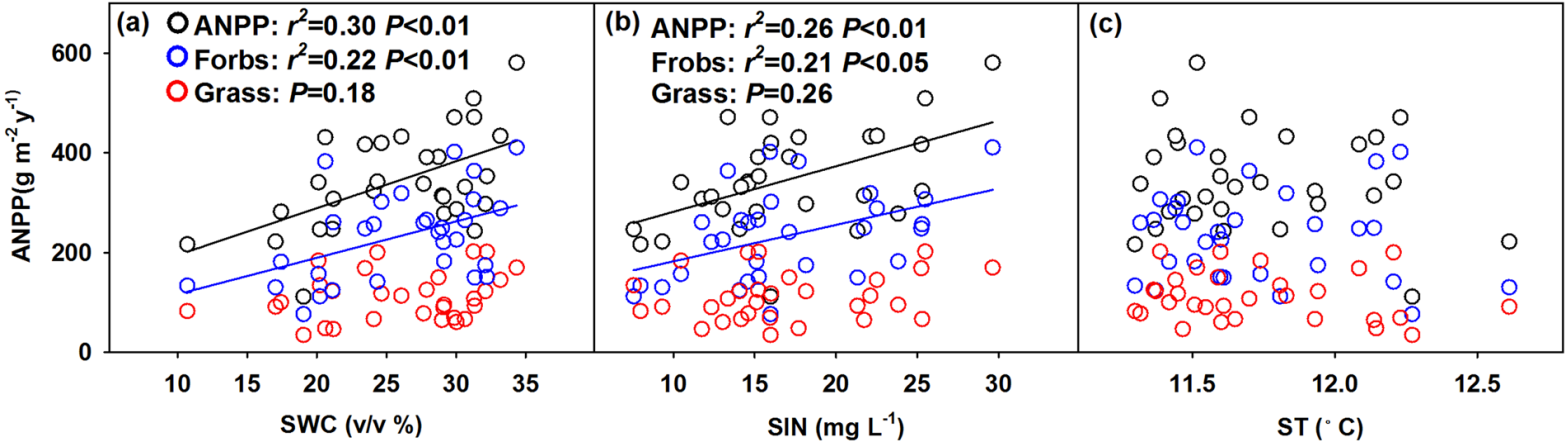
Relationships of aboveground net primary productivity (ANPP) with soil water content (SWC) (**a**), soil inorganic nitrogen concentration (SIN) (**b**) and soil temperature (ST) (**c**) in 2016.

The variations of ANPP in 2016 showed positively linear correlation with SWC (*P* = 0.002; Fig. 5a) and SIN (*P* = 0.004; Fig. 5b) across plots, whereas no significant relationship was found between ANPP and ST (Fig. 5c). Linear regression analyses demonstrated that SWC and SIN explained 29.97% and 26.37% of the variation in ANPP, respectively. The two factors together could explain 37.00% of changes in ANPP based on the multiple regression analysis (*P* < 0.01). Unlike grasses, productivity of forbs was sensitive to SWC and SIN, which increased linearly with increasing of SWC and SIN (Fig. 5a,b). SWC and SIN contributed to 22.26% and 20.74% of the variation in forbs biomass, respectively.

## Discussion

This study shows how much precipitation is extreme enough to cause a threshold response of ecosystem productivity. The threshold of precipitation for productivity was proposed in previous studies, but it lacks of empirical evidence^45–47^. In this study, we found a significant decrease in ANPP (*P* = 0.014, Fig. 2d) under 1/12 P treatment in 2016, which quantified the precipitation threshold of ANPP under extreme dry conditions. The nonlinear response of ANPP to precipitation gradient suggests that ANPP will decline strongly in extreme dry conditions, which presents as a negative asymmetric response at extreme low precipitation. The nonlinear relationship was inconsistent with the linear ones commonly reported in previous studies^11–13^. For example, in another manipulative experiment that includes three levels of rainfall reduction (30%, 55%, and 80%) in the Patagonian steppe, the authors found significant linear relationship of ANPP with precipitation amount^15^. This may be due to that their treatments only cover the linear response stage and may not reach the threshold of the ecosystem. So far, more than 85 precipitation experiments have been conducted in the world^48^. Due to a narrow range of precipitation, these experiments rarely find the threshold or nonlinear relationship between ANPP and precipitation. This study, to our knowledge, is among the first shows the nonlinear response of ANPP to precipitation gradient by using a manipulative experiment^17^. It partly supports the double asymmetric hypothesis proposed recently by Knapp, *et al*.^16^, and enriches the current understanding on the precipitation-ANPP relationship.

Other treatments hardly affect ANPP, which can be explained as follows. First, plant may reduce stomatal conductance and contents or activities of photosynthetic enzymes to adapt to moderate drought, resulting in mild reduction of ANPP instead of abrupt collapse of ecosystem^49^. Second, deep soil moisture storage from groundwater, snow accumulation and ablation in the high Zoige Basin may partly compensate the depletion of surface water for plant growth^50,51^. Our findings also provide the time series of the dynamic responses of ANPP to precipitation changes. Unlike the significant reduction in 2016, ANPP showed no significant differences among treatments even under 1/12 P treatment in 2015. This was probably because the lagged effect of precipitation from 2014 or even before. A previous study demonstrated that current-year production is determined by previous-year precipitation^52^. The findings indicate that both drought intensity and duration substantially affect ANPP responses to precipitation change.

A significant increase was found in BNPP at the depth of 20–40 cm under 1/12 P and 1/4 P treatments (Fig. 3b), suggesting that plants could allocate more biomass to deep soil to capture the limited resources in order to maximize their growth rate^53^. Since SWC at the depth of 10 cm decreased dramatically under 1/12 P and 1/4 P treatment, more biomass was allocated to deeper roots to absorb deep soil water. Although BNPP at the depth of 20–40 cm increased, there was no significant difference of BNPP at 0–40 cm between treatments because BNPP at 20–40 cm only accounted for 11.54% of total BNPP on average and BNPP at 0–20 cm didn’t change with precipitation treatments. Previous studies reported contradictory results on the responses of belowground biomass to precipitation change, with an increase or a decline of root biomass under drought condition^54,55^, which may be due to the various drought intensity and duration among studies. For example, moderate water stress with 51-day treatment can enhance root productivity by a surplus of assimilates that are exported to the roots due to allocation changes^55^. Whereas a ten-year drought treatment significantly diminishes BNPP^54^. Moreover, different edaphic and climate conditions between sites also contribute to the differential BNPP response to drought^56^. In line with our findings, the lack of response of root productivity and biomass to precipitation gradient was reported in temperate grasslands as well^12^.

Root productivity and biomass are determined by the dynamics of root growth and root death. Root growth of a plant is determined by carbon allocation to BNPP vs. ANPP (i.e., BNPP: ANPP ratio) while root death is related to root turnover times. The lack of response of root productivity or biomass to drought was probably due to an increase in the proportion of carbon allocation to roots and a decrease in turnover of roots with decreasing precipitation^57,58^. The rising trend in root/shoot ratio under drought may facilitate greater water capture and thus optimize root growth under a dry environment (see the detailed discussion next paragraph). It is proved also by the increasing BNPP at 20–40 cm under extreme drought treatment (Fig. 3b). Some studies also confirmed that many new roots are long and slender under drought conditions^59^. Root turnover rate was not monitored in this study, but previous studies demonstrated a reduction of root turnover with decreasing precipitation^60^. In all, compared with ANPP, BNPP has more uncertainty under precipitation changes. Additional studies on the mechanism underlying the effect of precipitation on dynamics of root growth and mortality are needed for better understanding of BNPP changes.

In spite of no significant differences of *f*_BNPP_ among treatments, the 1/12 P plots tended to have higher *f*_BNPP_ than other treatments (Fig. 3c). This was probably a consequence of plant adaptation to extreme dry condition by regulating proportion of the biomass allocation toward belowground. Some previous studies confirmed that plants increase *f*_BNPP_ to optimize growth under drought conditions, likely resulted from changes in the relative importance of limiting resources (such as water, light, nutrients)^12,34^. However, some other studies stated *f*_BNPP_ is not influenced by water supplementation^61^. Although the mechanisms behind the allocation shift under drought are unclear, the decline tendency of *f*_BNPP_ with increasing precipitation (Fig. 3c) supports the optimal partitioning theory and provides important constraints for the calibration and testing of dynamic carbon cycle models.

SWC has been proposed to be an important index in forecasting ecosystems’ responses to climate change^62,63^. The positive linear correlation between ANPP and SWC in 2016 suggests that SWC can better predict the variation in ANPP than precipitation amount. Comparing with precipitation amount, SWC are more responsible to ANPP changes, which can be attributed to the following two reasons. First, although growing season precipitation amount was recognized as a predictor of ANPP in grassland, soil moisture directly links to root activity, plant water status, and photosynthesis in physiology^32,64^. Other soil resource availability is also chronically altered through soil water dynamics^65^. Second, SWC was mediated by the water-storage capacity of the soil, which is better than precipitation to express water availability for plant growth^66^.

We also found that SIN explained 26.37% of the variation in ANPP across plots under different precipitation treatments (Fig. 5a). Because rainfall is the primary source of new nitrogen inputs to the system by net deposition and soil moisture also impacts soil nitrogen mineralization by changing the structure and function of soil microbial communities^67^, precipitation changes largely alter SIN dynamics. The reduced N availability under dry condition would constrain plant N uptake and growth, leading to lower productivity^68,69^. In addition, previous studies also indicated that total inorganic nitrogen is linearly related to natural annual precipitation^70^. Therefore, in the study site of alpine meadow where SIN limits plant production^71^, precipitation effects on ANPP are partly attributable to changes in SIN. The direct effects of soil water availability and the indirect effect through SIN in combination largely explained the ANPP variation across treatments. Our findings highlight SIN changes should be taken into consideration in understanding and modeling ANPP response to altered precipitation.

Beside the abiotic effects, biotic impacts of species composition also influence ANPP responses to precipitation change. As a major proportion of community (>67%), forbs biomass reduced significantly under extreme drought in this study, which led to an abrupt drop in ANPP (Fig. 2f). It was more sensitive to precipitation changes and more inhibited by extreme drought, because the growth of forbs usually requires more water than grasses^72^. Consequently, we predict that shifting species composition toward less sensitive species may dampen the response of ANPP to precipitation change.

## Methods

### Study site

The study was conducted in an alpine meadow located in Hongyuan county (32°48′N, 102°33′E, 3500 m a.s.l.), which is in the eastern of Qinghai-Tibetan Plateau. The mean annual temperature is 1.5 °C in the study site over the past 50 years. The average temperature of the hottest month (July) is 11.1 °C, and the mean of the coldest months (January) is −9.7 °C. The mean annual precipitation is 747 mm. The meadow community at our experimental site is dominated by grasses of *Deschampsia caespitosa*, *Elymus nutans*, and *Agrostis hugoniana* and forbs of *Anemone rivularis, Potentilla anserina*, and *Polygonum viviparum*. The soil of the study is classified as Mat Grygelic Cambisol according to Chinese Soil Taxonomy Research Group^73^, with mean bulk density is 0.89 g cm^−3^_._

### Experimental design

**Figure 6:**
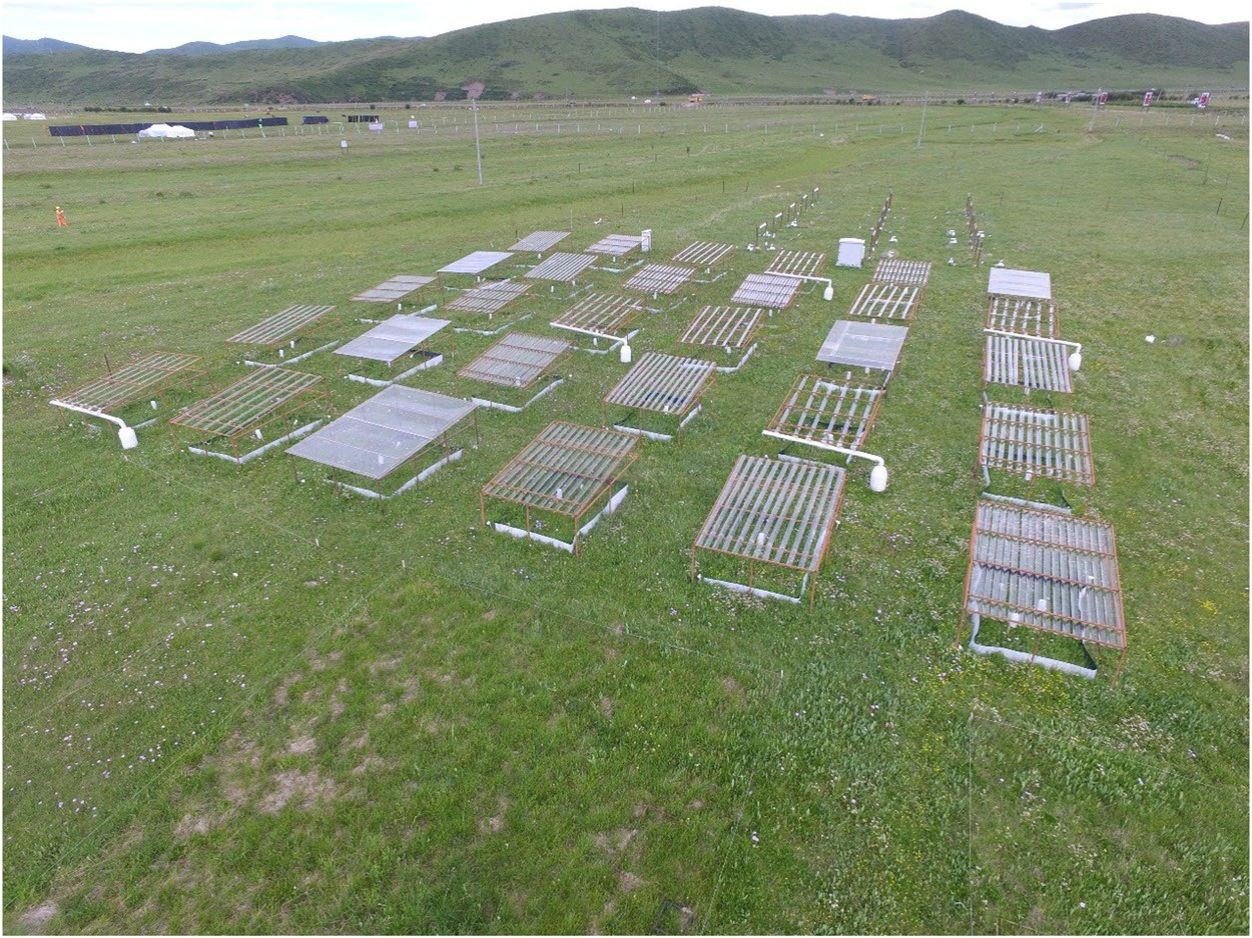
Plot layout and experimental design of the study. The varying levels of precipitation are achieved using combinations of water catchment and rainout shelters.

The precipitation treatments have been conducted from May, 2015. It used a randomized complete block design with six levels of precipitation (1/12 P, 1/4 P, 1/2 P, 3/4 P, P and 5/4 P, P is the annual precipitation). Each treatment was replicated five times, and each replicate plot was 2 m × 1.5 m. The experiment consisted of thirty plots in six rows, with 2 m between the rows and between plots within a row (Fig. 6). We achieved the varying levels of precipitation using combinations of water catchments and rainout shelters. The rain-shelter was used to reduce precipitation as described by Yahdjian and Sala^74^, which is a fixed-location shelter with a roof consisting of curved bands of transparent acrylic that block different amounts of rainfall while minimally affecting other environment variables. Each shelter has a fixed metal structure (4 m in length, 3 m in width, 1.0–1.5 m in height). To minimize disturbance, we mechanically pushed fiberglass plats down to a depth of 40 cm in the soil surrounding the plots as in the Jasper Ridge Global Change Experiment^75^ to cut off lateral movement of soil water. The devices help achieve the goal of a free-air controlled experiment with minimal site disturbance. The 5/4 P treatment was made by adding water taken from the 3/4 P treatment. Under 3/4 P treatment, 1/4 P rainfall was accepted and removed from the plot. This gave us six precipitation levels without modifying the precipitation frequency and timing in our design.

### Measuring variables

#### Rainfall, soil water content, temperature, and inorganic nitrogen concentration

The exact rainfall received by each plot was measured by rain gauge settled in the middle of each plot at the height of 20 cm. The precipitation amount was computed right after each rainfall event. Soil water content (SWC) and temperature (ST) in the top 10 cm were measured using a portable Time Domain Reflectometry equipment (TDR 100, Spectrum Technologies Inc., Chicago, USA) and sensors of LI-6400–09 (LI-COR Inc., Nebraska, USA), respectively, once a week over the growing season in both 2015 and 2016. Soil samples were collected at the end of the growing season, sieved through a 2 mm mesh. A subsample of 10 g of soil samples was extracted for measurement of inorganic nitrogen (NH_4_^+^ and NO_3_^−^) in 50 mL 2 mol/L KCl on a rotary shaker for 1 h within 24 h. The filtrate made using filter paper was analyzed using the AA3 Continuous Flow Analyzer (AA3, SEAL Analytical GmbH, Germany).

#### ANPP, BGB, BNPP measurement and *f*_BNPP_ estimation

ANPP was directly measured by clipping the sample strip (0.12 × 1.00 m) in each plot at peak biomass stage in each year (usually in the early of August). We separated the samples into different species, oven-dried at 65 °C for 48 h, and weighed. BNPP was measured by ingrowth core method^34,76,77^. Soil cores (diameter 9 cm) were taken from the same spot in each plot, with two soil layers (0–20 cm, 20–40 cm) at the peak biomass of vegetation in 2015. The holes were immediately filled with sieved root-free soil originating from the same depth outside of the plots that contained similar soil profile properties as the sampled ones. After one year, the soil cores of the same holes were taken with a soil auger of 7.5 cm diameter at the two layers. Different depths of soil cores were transferred into plastic bags and washed by filter (0.25 mm) under smoothly flowing water to obtain the root samples, oven-dried at 65 °C for 48 h, and weighed to the nearest 0.01 g. Belowground biomass (BGB) was measured using the roots of 2015, BNPP was estimated by the samples of 2016^30^.

$${f}_{{\rm{BNPP}}}{\rm{wascalculatedas}}{f}_{{\rm{BNPP}}}=\mathrm{BNPP}/({\rm{ANPP}}+{\rm{BNPP}}).$$

### Statistical analysis

One-way ANOVA was performed to analyze the differences of ANPP, BGB, BNPP and *f*_BNPP_ among the treatments in each year. Stepwise multiple linear analyses and nonlinear regression analyses were used to evaluate the relationships of ANPP, BGB and BNPP with PPT, SWC, SIN and ST. All statistical analyses were conducted with SPSS 19.0 software (SPSS Inc., Chicago, IL, USA).
